# Sexual dimorphism and morphological integration in the orchid bee brain

**DOI:** 10.1038/s41598-025-92712-3

**Published:** 2025-03-14

**Authors:** Denise Yamhure-Ramírez, Peter C. Wainwright, Santiago R. Ramírez

**Affiliations:** Department of Evolution and Ecology, University of California, Davis, CA 95616 USA

**Keywords:** Allometry, Integration, Hymenoptera, Neuroethology, Neuropil, Animal behaviour, Sexual dimorphism

## Abstract

Sex-specific behaviours are common across animals and often associated with sexual dimorphism in the nervous system. Using micro-CT scanning we standardized sex-specific brain atlases and tested for sexual dimorphism in the brain of the orchid bee *Euglossa dilemma*, a species with marked sex differences in social behaviour, mating strategies and foraging. Males show greater investment in all primary visual processing neuropils and are uniquely integrated with the central complex, evidenced by a strong positive covariation. This suggests that males invest more on locomotor control, flight stability and sky-compass navigation which may have evolved in response to sex-specific behaviours, like courtship display. In contrast, females have larger mushroom bodies that strongly and positively covary with the optic lobes and have increased volume of the Kenyon cell cluster, implying greater capabilities for visual associative memory. We speculate this is an adaptation to social and nest-building behaviours, and reliance on learning visual landmarks required for central place foraging. Our study provides the first record of sexually dimorphic morphological integration in the brain of an insect, an approach that revealed sex-specific brain traits that lack an apparent morphological signal. These subtle differences provide further evidence for the causal link between brain architecture and behaviour.

**Supplementary Information:**

The online version contains supplementary material available at 10.1038/s41598-025-92712-3.

## Introduction

Animals typically exhibit sex-specific behaviours, and as a result the nervous system— particularly the brain—is prone to evolve sexually dimorphic traits. The form and function of this complex organ often varies in response to environmental selective pressures that arise from the behavioural differences between the sexes^1^. Dimorphism in the brain occurs in morphology, gene expression, neural circuitry, hormone levels, and other traits^2^.

Insects offer excellent opportunities to explore how behavioural differences correlate with sexual dimorphism in the brain, as their lifestyle often correlates with variation in different brain regions^3^. These regions, known as neuropils, are characterized by their associated sensory input and neural processing^3,4^. The differential volumetric expansion of these neuropils is often associated with sensory or behavioural adaptations and indicate changes in either neuron number/size and/or connectivity, suggesting changes at the functional level^5^. For example, neuroanatomical sexual dimorphism has been clearly documented in mosquitos, in which females show an enlargement of the visual and chemical processing neuropils, while males have enlarged hearing centres. This dimorphism in the brain has been attributed to their sex-specific reproductive biology, where females must locate a host, while males use the wingbeats of females as auditory cues to locate mating swarms^6^.

Invertebrates are excellent examples of how relatively small brain can produce a variety of complex behaviours that require a high degree of cognitive abilities, emphasizing that it is often the underlying neural functions and not the absolute mass of nervous tissue that matters^7^. For these reasons, it is important to understand the limitations of exclusively volumetric comparisons, even at an intraspecific level, and complement this approach with methods that instead attempt to measure interconnectivity between brain regions.

Variation of morphological traits can be used to quantify morphological integration between different phenotypic structures of an organism. Morphological integration, measured by covariance between traits, refers to the way different traits of an organism become linked through developmental processes and organismal function^8–10^. In the insect literature, morphological integration has been used to study shape covariation to infer functional traits. For example, Barden et al. (2020) showed how the unique integration between the head capsule and the dorsoventrally expanded mandibles of the extinct cretaceous hell ants (Haidomyrmecine) provided the functional basis of their specialized predation. Morphological integration has been less explored in more inconspicuous traits, such as the brain [see^11,12^], and has never been compared between different sexes of the same species. Our approach measures how the different brain regions differentially expand while we also consider pair-wise covariations between neuropil volumes. Assessing morphological integration therefore provides a novel way to reveal a shared functionality between structures, and by extension, help inform the relationship between brain anatomy and behaviour.

The orchid bee *Euglossa dilemma* is one of the few orchid bees with extensive literature on life history, behaviour, and evolution, making it an excellent species to identify correlates between brain anatomy and behavioural traits. This neotropical pollinator exhibits strong intersexual differences in foraging behaviour and ecology, as the sexes differ in the resources they need for reproduction.

Females of *E. dilemma* are mass-provisioning and primitively eusocial, forming small cooperative colonies of usually one or two subordinate daughters and one dominant mother that passes through a solitary phase before moving into a social phase^13^. Importantly, these social interactions often involve the same individual bees and typically span months with the same individuals interacting with each other. Moreover, female bees will only mate once and will typically nest in a single location for their entire life while exhibiting central place foraging for food and nesting materials (i.e., nectar, resin and pollen)^13,14^. This behaviour is known to require visual associative memories to determine spatial relationships through visual landmarks^15^.

Contrary to females, males of *E. dilemma* are nomadic and solitary, leaving the maternal nest soon after emergence. They will collect chemical compounds (i.e., oils) from the environment to concoct a species-specific perfume which they use as a sexual signal during courtship^16^. These perfume mixtures are obtained by visiting various environmental sources, including fungi and orchid flowers, which make them energetically costly^17,18^. These chemical signals evolved by sexual selection through female choice and male-male interactions^19^. This behaviour is associated with wider foraging ranges and limited site fidelity as it requires the visitation of multiple sparsely distributed resources^17,20,21^, potentially requiring a different set of cognitive demands for navigation. Males do exhibit sporadic male-male competitive interactions where they will aim to steal another male’s perfume, those interaction often involve short interactions that span a few minutes and typically involve different individuals, thus not requiring the same cognitive abilities of social female bees.

We use micro-CT scanning with a novel non-destructive methodology for soft-tissue enhancement to study and describe in detail the sex-specific brain architecture of newly emerged *E. dilemma*. We tested for dimorphism in allometric scaling and relative sizes (as a proxy of investment), and further asses morphological integration between the brain regions to study their strength of interaction and independence. A previous study by Brand et al. (2018) showed sexual dimorphism in visual and olfactory centres of *E. dilemma*, however, our study differs in four main ways. (1) We control for effects of age and/or experience, both factors known to affect the volume of different brain regions by processes of neuroplasticity. (2) We evaluated more brain regions for a more precise understanding of the sensory difference between the sexes. (3) Our methods to assess relative investment use standardized major axis regressions instead of direct measurements of relative volumes, which allowed us to account for the differences in the allometric scaling across the neuropils here studied. (4) We measured morphological integration, an approach not used by Brand et al. (2018).

We identify differences in brain regions that correlate with the behavioural ecology of each sex. Our results show that males invest more in all primary visual processing centres, including those areas specialized in processing polarized light, and their central complex positively covaries with the optic lobe. We also show that females possess larger mushroom bodies with an increased number of intrinsic neurons which positively covaries with two optic neuropils: one involved in chromatic representation and the second in shape detection. This study offers the first record of sexually dimorphic morphological integration in an insect brain and shows how this sex-specific variation in morphological traits may support the sensory and cognitive demands of the sexes under an ecological framework.

## Results

### Sexual dimorphism in brain regions shows association to ecological and behavioural differences

We built a sex-specific brain atlas of newly emerged *E. dilemma* male and female bees, to control for any post-eclosion growth caused by neuroplasticity. Each atlas includes nine main region which were recognizable given differential contrast (e.g., lateral horn), and/or discrete structures (e.g., medulla), and have reported known functions in different insect species (Table 1), as well as the remaining central brain (rCB).

**Table 1:** Reported functional profile of the neuropils studied.

Brain Region	Function	References
Lamina	Primary processing of visual information from the compound eye- local motion computation, determination of the contour through light-dark contrast, processing of polarized light	^22–25^
Medulla	Primary process of visual information from the compound eye - spectral opponency as well as inhibitory and excitatory responses to specific wavelength, first full chromatic representation sent to high-order processing, processing of polarized light	^24–26^
Lobula	Primary process of visual information from the compound eye - phasic and tonic colour opponency, non-colour opponent cells computing brightness of the environment	^24^;^27,28^
Anterior optical tubercle + tract	Secondary unimodal processing of visual information- Targeted by the medulla and lobula, it is involved in chromatic and colour-opponent processing, as well as processing of polarized light for polarization-based compass	^29^;^30^
Ocellar synaptic plexi	Primary processing of visual information for them ocelli-, detection of changes in light intensity, flight stability	^31^;^32^
Antennal lobes	Primary processing of olfactory information- olfactory coding with recognition and discrimination of odours.	^33,34^
Lateral horn	Secondary unimodal processing of olfactory information- posterior processing of the olfactory coding through feedforward inhibition and segregation to encode preferences for innately meaningful odours.	^35,36^
Mushroom bodies: Calyx, pedunculus and lobes	Multimodal high-order processing centre composed by Kenyon cells, the centre’s intrinsic neurons - context generalization, behaviours of temperature preference, conspicuous orientation and sleep, associative learning, long and short-term associative memory. **Calyx**: Input region of the mushroom body, composed of the dendrites of the Kenyon cells **Lobes**: alpha and beta- main sensu-lato output sites and memory consolidation **Pedunculus**: Axon formed stalk-like structure connecting the calyx to the lobes	^37–40^
Central complex: protocerebral bridge, fan-shaped body, ellipsoid body and noduli	High-order processing – Centre of locomotor control for navigation, coding of celestial compass cues	^41–43^

**Fig. 1:**
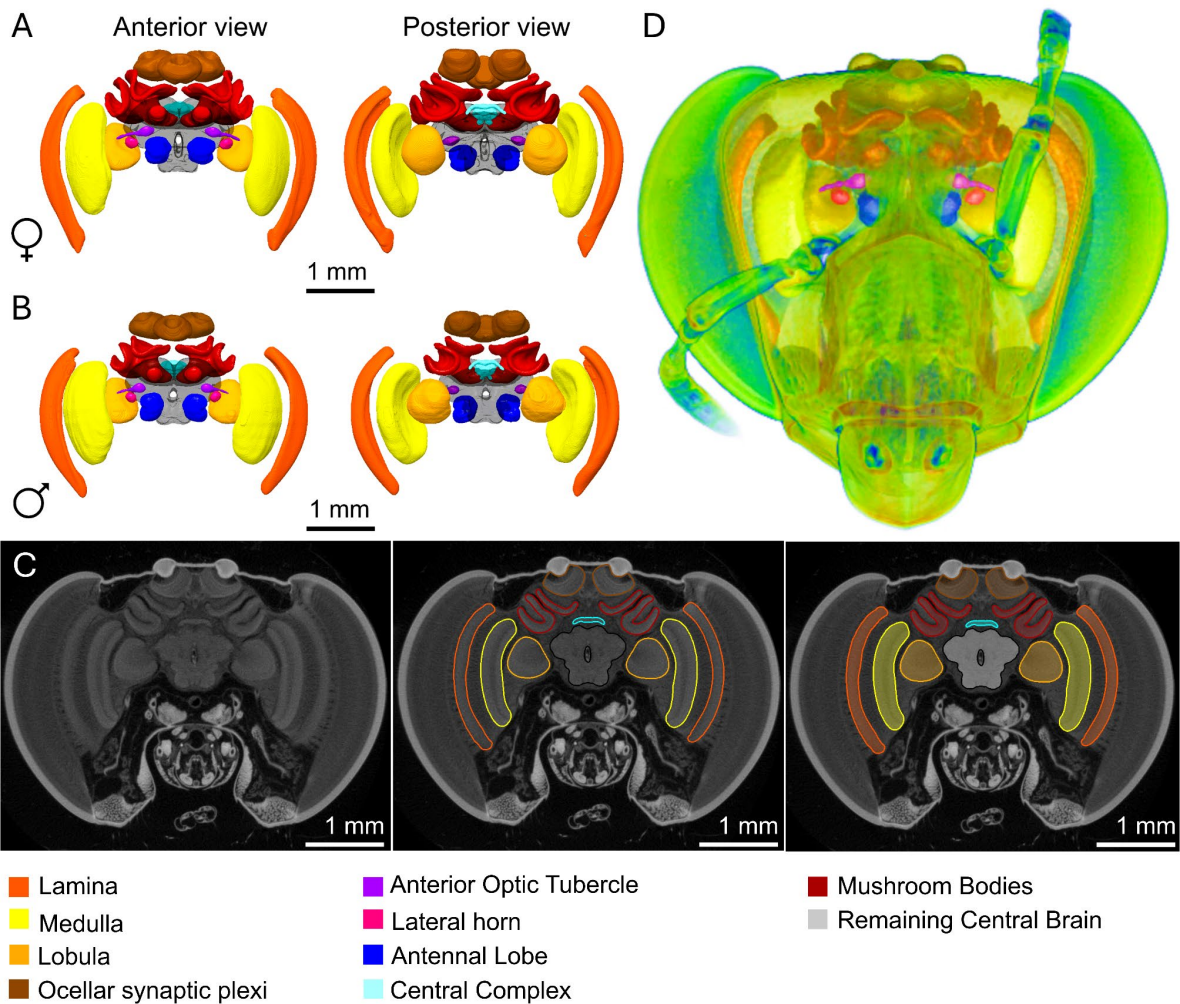
Workflow for micro-CT 2D data analysis and 3D image visualization 3D reconstruction of the standardized brain atlas of (**A**) female Euglossa dilemma (*n* = 13) and (**B**) male *Euglossa dilemma* (*n* = 12) a, shown from the anterior and posterior view. The colour code at the bottom indicates the different segmented neuropils (see also Table 1). (**C**) Representation of a single micro-CT slice from a female *E. dilemma* stained with I_2_ for neural tissue enhancement, and the subsequent segmentation process (**D**) 3D visualization of the reconstructed *E. dilemma* female’s brain residing in its natural spatial arrangement within the head capsule.

This atlas reveals pronounced differences between the sexes. For example, males have a more vertically constricted arrangement which is compensated by a wider volume on the sagittal plane (Fig. 1A, B). These differences are due to the head morphology of each sex, in which females have significant longer (*p* < 0.05) and thinner (*p* < 0.001) faces compared to males (supplementary Figure S1). Observing these subtle differences in brain shape and morphology was possible thanks to the staining protocol used, which was done without making an incision in the head capsule and thus allowed visualization of the intact brain and its natural spatial arrangement (Fig. 1C, D).

**Fig. 2:**
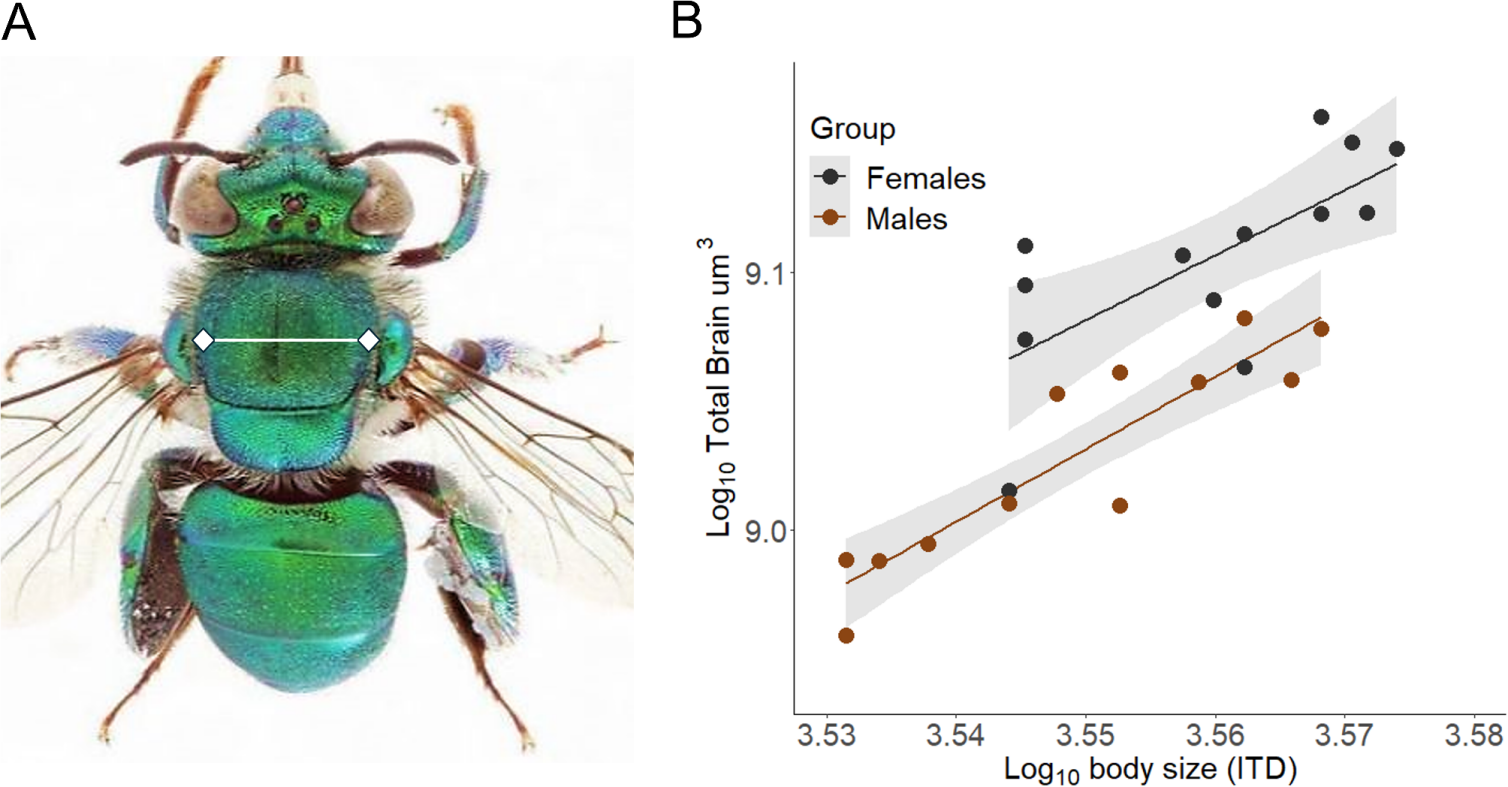
Body-brain scaling relationship is shared between the sexes (**A**) Male *E. dilemma* illustrating how the intertegular distance (ITD), indicated as white line, was measured as a proxy of body size. Photo by Joseph Wilson. (**B**) The relationship between total brain size and body size follows an isometric scaling in both sexes, with females tending to be larger than males indicated by a slight sift along the x-axis and possess relatively larger brains indicated by the grade-shift in the y-axis.

Scaling relationships within a biological system help maintain functional equivalence and in *E. dilemma* we found that the brain maintains a proportional relationship with body size. As the total brain size was measured as a volume (µm^3^), and body size as a linear measurement (the intertegular distance, µm) (Fig. 2A), after accounting for the different dimensionalities we found that both sexes share a common slope between the total brain size and body size, which does not deviate from isometry (shared β = 3.1, *p* > 0.5, Fig. 2B). This result is consistent with previous studies of other hymenopteran insects of similar size, where the relationship between body size and brain size is isometric^44^, counter to the prediction described by Haller’s rule, which states that relative brain size should decrease with increasing body size^45^. However, we found that females have a relatively larger brain, indicated by a higher Y-intercept in the regression line (*p* < 0.001, Wald χ2 = 16.8; Fig. 2B).

In addition to the shared overall body-brain scaling, the sexes share volumetric scaling patterns between distinct neuropils to overall brain size when using the remaining central brain as an independent measure of brain size, with a strong effect size while using Cohen’s categorization (see Materials and Methods). The scaling of the different brain regions is in many cases isometric, following the overall patterns described in insect brains^46^. However, the antennal lobes as well as the two high-order processing centres, the central complex and the mushroom body, deviated from isometry (slope.test = 1, *p* < 0.05) and instead shared negative allometry between sexes, with brain regions smaller than expected in a larger brain, with an average β of 0.6, 0.61 and 0.54 respectively. The negative allometry of the mushroom body held true when analysing the calyx and the pedunculus and lobes (alpha and beta) regions independently (see Table 1 for reference), as well as the visual and olfactory compartments of the calyx, known as the collar and lip^40^. Of all the brain regions considered, the ocellar synaptic plexi was the only one exhibiting positive allometric scaling, with an average slope of 1.3. (see all summary statistics in supplementary Table S2).

**Fig. 3:**
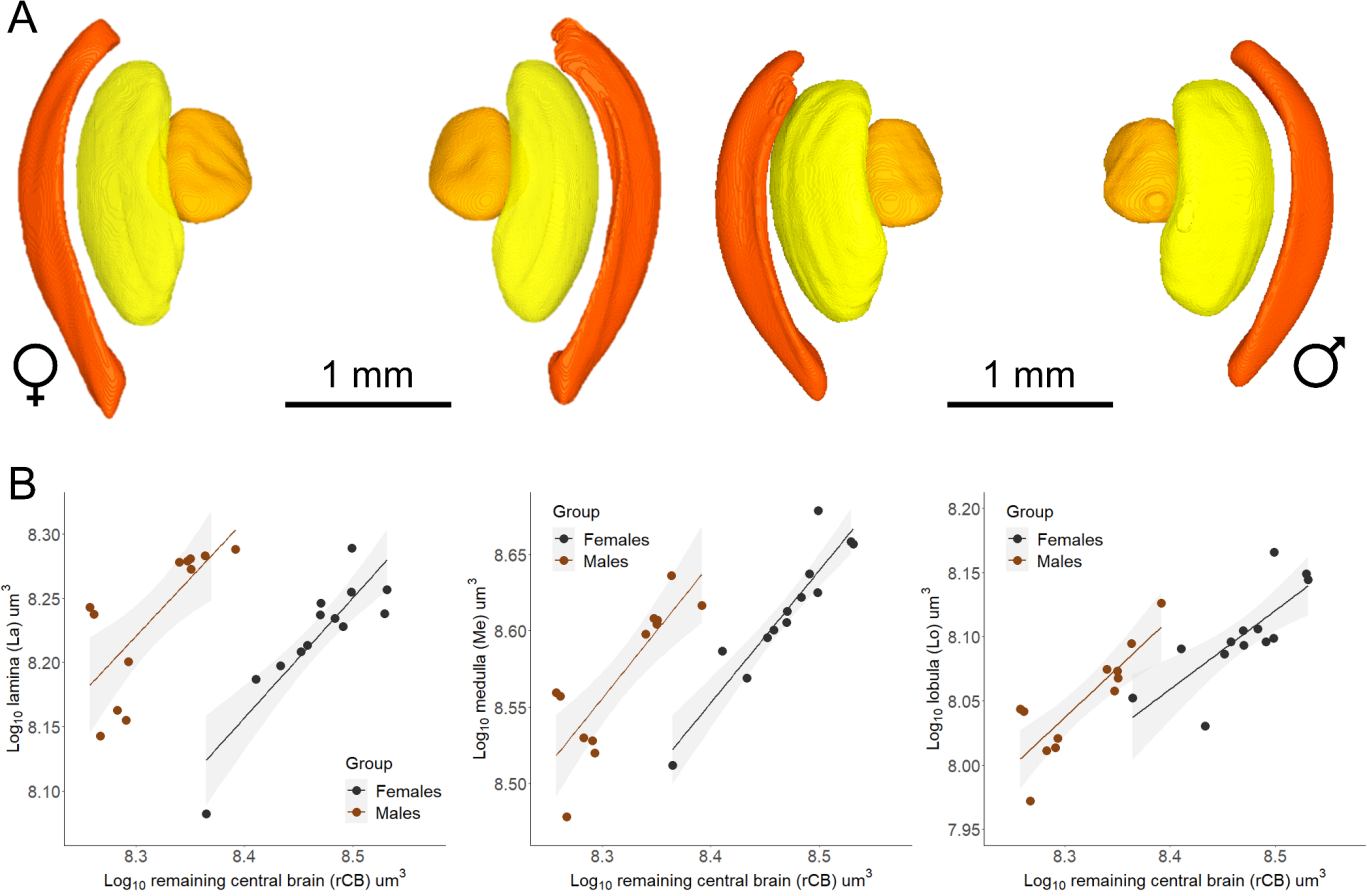
Sexual dimorphism in the optic lobe of *Euglossa dilemma*, with a relative 1.3 times enlargement in males. (**A**) 3D visualization of the reconstructed lamina (orange), medulla (bright yellow) and lobula (mustard yellow), forming the optic lobe of *Euglossa dilemma* females (left) and males (right). (**B**) With an isometric relationship with the remaining central brain, the three neuropils of the optic lobe show an upshift in their relative volume compared to females. With an increase of 1.5 X in the lamina, 1.3 X in the medulla and 1.2X in the lobula, males invest on average of 1.3 times more in this primary centre of visual processing (See also supplementary Tables S2, S3).

Males and females exhibit sexual dimorphism in the relative investment (i.e., sizes) of specific neuropils. The most pronounced difference is in the optic lobe (Fig. 3A), known to be the primary processing centre of visual information from the compound eyes. This lobe is composed of three distinct neuropils known as the lamina, medulla and lobula (see Table 1 for functional profile). Males invest an average of 1.3 times more in each region compared to females (relative lamina volume -Males: 0.146 ± 0.007; Females: 0.119 ± medulla volume- Males: 0.315 ± 0.009; Females 0.292 ± 0.005, lobula volume- Males: 0.095 ± 0.003; Females 0.090 ± 0.004, *p* < 0.0001, Fig. 3B, supplementary Table S3).

**Fig. 4:**
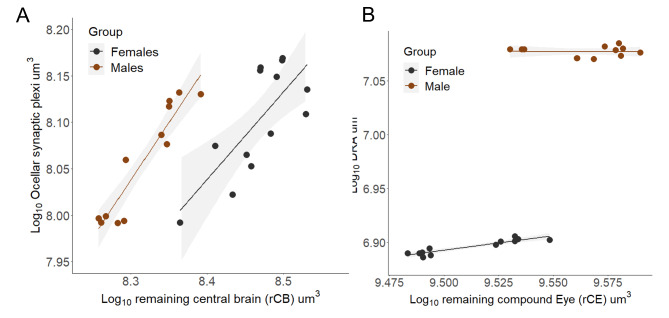
*Euglossa dilemma* males present enlarged visual regions involved in polarized light-based navigation. (**A**) Isometrically scaling with the remaining central brain, the ocellar synaptic plexi is sexually dimorphic, with an upshift in its relative size seen in males. (**B**) Controlling for the size of the compound eye and with a shared negative allometry between the sexes, males have a larger dorsal rim area.

In addition to a larger optic lobe, males have relatively larger specialized structures for processing polarized light. The ocellar synaptic plexi, formed by the nerves from the three ocelli – light capturing eyes–^31^;^32 ^is 1.3 times larger in males (relative ocellar plexi volume -Males: 0.097 ± 0.005; Females: 0.090 ± 0.006, *p* = 1.63-06, Fig. 4A, supplementary Table S3). Additionally, the dorsal rim area (DRA), located in the dorsal-most margin of the compound eye and recognizable in the micro-CT scans as a protrusion with a thinner cornea (supplementary Figure S4), is larger in male (Fig. 4B). The cornea is the chitinous lens of the compound eyes that can be distinguished in the scans as a white bright layer in the outermost part of the eye. As the cornea is not soft tissue, which requires contrast for visualization, the iodine enhances the x-ray absorption making it look brighter than the rest of the structures of the eye. In both sexes, this small region, known to facilitate polarized light-based navigation^47^, scaled with strong negative allometry to the rest of the compound eye, with an average slope of 0.29.

**Fig. 5:**
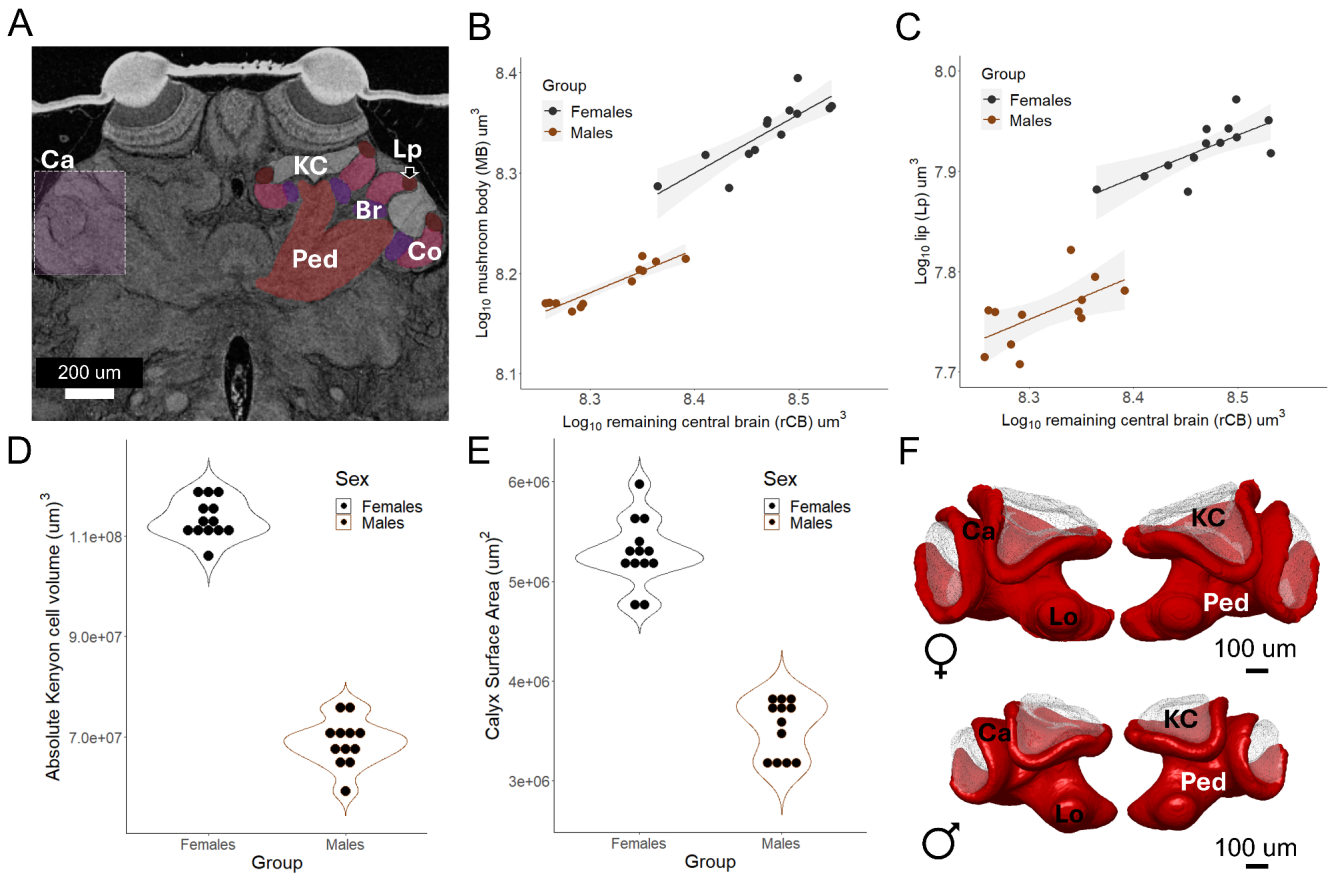
The mushroom body- the centre for learning and memory - shows increased size and neuron number in *E. dilemma* females. (**A**) Single micro-CT slice showing the regions of the mushroom body. The calyx (Ca) is the input region (light pink box) with three subcompartments that receive sensory-specific information. The lip (lp) for olfactory input (dark red), the collar (Co) for visual input (pink), the basal ring for multisensory information. The intrinsic neurons- Kenyon cells (KC) -(white) reside in and around the calyx. The pedunculus and lobes are the sensu-lato output region (red) (**B**) With a shared negative allometry females show an upshift in the overall size of the mushroom body compared to males (see supplementary Table S3). (**C**) The expansion of the calyx in females is driven primarily by an upshift in the lip. (**D**) Total volume of the Kenyon cell cluster as a proxy of neuron number, showing a larger cluster in females. (**E**) Interpolated surface area of the calyx with increased surface on females. (**F**) 3D average reconstruction of the mushroom body in female and male *E. dilemma* (red) and associated cluster of Kenyon cells (transparent white-black dotted), showing the association between surface area and volume of neuron cluster.

We also identify sexual dimorphism in the mushroom bodies, which are the primary centre for associative learning and memory consolidation in insects^37,48,49^. The mushroom body can be divided into three main substructures, known as the calyx, pedunculus and lobes (see Table 1 for functional details), which are important to differentiate given their functionality as discrete input, output and memory consolidation structures, respectively^37–40^. Our results show that all three regions show differential expansion in females, with a prominent increase in the volume of the calyx’s olfactory sub-compartment known as the lip (Fig. 5A, B, C, supplementary Table S2). The mushroom body is 1.2X larger in females (relative mushroom body volume -Males: 0.130 ± 0.007; Females: 0.156 ± 0.005, *p* = 1.22-15) and the coefficient holds for the expansion of each region independently (supplementary Table S3). The expansion of the mushroom bodies correlates with a significant increase in the cluster of Kenyon cells (Mann-Whitney U test *p* < 0.0001). Kenyon cells are the intrinsic neurons that form this neuropil, extending dendrites that form the calyx in which they reside, and axons that form the pedunculus and lobes^38^ This suggests that the expansion of the mushroom body was not driven by a larger dendritic growth of a common number of neurons shared between the sexes, but rather an increment in the number of neurons forming the neuropil (Fig. 5D). The enlarged cluster of cells was supported with a larger surface area of the calyx, which in aculeate hymenopterans has evolved into a more elaborate morphology with deeply curved cups that increase the surface-area-to-volume ratio where the Kenyon cells reside (Fig. 5E, F).

### Morphological integration across brain regions is sexually dimorphic

**Fig. 6:**
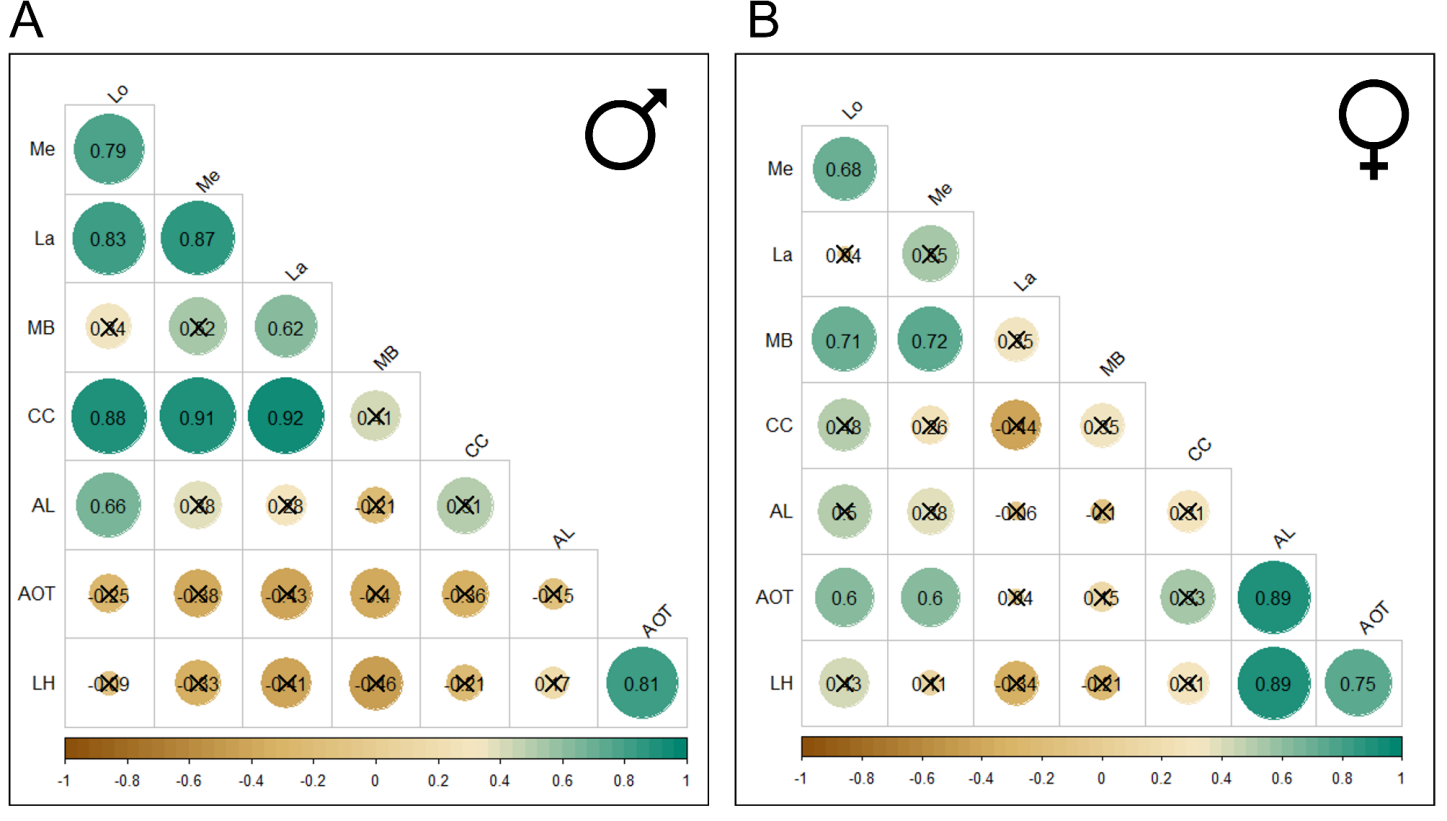
Morphological integration between neuropils exhibits a sexually dimorphic pattern. Correlation matrix showing the strength of morphological integration between neuropil in the brain of (**A**) *Euglossa dilemma* males and (**B**) *Euglossa dilemma* females. The colour bar represents the strength of the correlation, which ranges from negative 1 to positive 1. Each pair-wise correlation displays a colour within this gradient that is equivalent to the value of the correlation. Most correlations are non-significant, indicating some independence from other brain regions and are shown as crossed values. Neuropils are labelled with the following abbreviations: antennal lobes (AL), anterior optic tubercle (AOT), central complex (CC), lamina (La), lateral horn (LH), lobula (Lo), medulla (Me), mushroom body (MB).

To investigate which brain region exhibits morphological integration, we conducted a correlation analysis with Pearson correlation coefficients among all pairwise comparisons. We found that the patterns of correlation vary strongly within each sex and between sexes (Fig. 6). For example, the neuropils of the optic lobe are highly integrated in males and each of them show strong integration with the central complex, a high-order processing centre for spatial navigation^41^ (see Table 1 for further references). These correlations among optical lobe neuropils were strong according to Cohen’s categorization (see materials and methods), with all values above 0.75 (lamina-medulla: *r* = 0.87, *n* = 12, *p* = 2.70e-04, lamina-lobula: *r* = 0.83, *n* = 12, *p* = 8.58e-04, medulla-lobula: *r* = 0.79, *n* = 12, *p* = 0.002), yet the integration with the central complex was even stronger, with the minimum correlation of 0.88 (Fig. 6A). This pattern of trait covariation is different in females, with the medulla and lobula only showing a signal of moderate integration between them (*r* = 68, *n* = 13, *p* = 0.01), and an absence of significant trait covariation with the lamina (medulla: *r* = 0.55, *n* = 14, *p* = 0.06, lobula: *r* = 0.04, *n* = 13, *p* = 0.9). These two visual neuropils are instead integrated with the mushroom body (medulla: *r* = 0.71, *n* = 13, *p* = 0.006, lobula: *r* = 0.71, *n* = 14, *p* = 0.005) and the anterior optic tubercle in females (medulla: *r* = 0.6, *n* = 13, *p* = 0.03, lobula: *r* = 0.6, *n* = 13, *p* = 0.031) (Fig. 6B). Additionally, the sexes differ in the integration between the primary and secondary centres for olfactory information, with the antennal lobes (primary) and the lateral horn (secondary) being strongly integrated in females (*r* = 0.89, *n* = 13, *p* = 0.000*04.2*6 e-5) and a non-significant integration in males (*r* = 0.17, *n* = 12, *p* = 0.59).

Our results indicate that only one pattern of integration between regions is shared between the sexes, namely the covariation between the lateral horn and the anterior optic tubercule. These two neuropils are the secondary processing centres for olfaction and visual information, respectively. This integration may be potentially driven by the small size of both neuropils and their physical proximity in the brain^50^. Besides the differences and singular similarity in integrated regions between the sexes, most correlations within each sex were non-significant (*p* > 0.05).

## Discussion

In insects, it is well documented how lifestyles pose selective pressures on the brain to produce adaptive behaviours that fit the ecology of an organism^3^.In this study, we identify sex-specific sensory adaptions in the orchid bee *Euglossa dilemma* that are present since the moment of the adult emergence, and are independent of post-eclosion neuroplasticity. These differences were expressed in both general morphology and integration and appear to be strongly associated with the marked behavioural differences and the sexually dimorphic niches of this species.

Associations between behavioural traits and the differential enlargement of specific brain regions at the intraspecific level have been well documented in animal models and are often associated with differences in reproductive strategies. For example, *Drosophila melanogaster* males show an enlargement in three glomeruli of the antennal lobes which are thought to be involved in male-male interactions and the detection of female pheromones^6,51^. In contrast, females have a larger optic lobe which is thought to be an adaptation to enhance perception of motion during odour plume tracking, a behaviour that strongly relies on the attraction to visual features that contrast with the environment^52^.

Our results highlight two primary sexually dimorphic trends in *Euglossa dilemma*. First, we show that males invest more in all primary visual processing regions which are uniquely integrated with the central complex (Figs. 3 and 6A). This dimorphic trait is likely to be a functional adaptation to the sensory demands of male reproductive biology, which involves foraging across larger home ranges for scent-collection and display behaviour, and interactions with other males at perch sites^19^). Our results agree with a previous report of sexual dimorphism in the relative volume of medulla of wild-caught *E. dilemma* bees^53^, yet it does not support the result that the absolute volume of the medulla is larger in males than females. This can be attributed to potential neuroplasticity processes occurring in the optic lobes of wild-caught females. Our study used newly emerged individuals, while the study by Brand et al. (2018) used wild-caught bees that varied in age, social experience and foraging. Transition from active lifestyles to in nest social habits, such as those found in social stages of *E. dilemma* females see^13^ can, in fact, lead to the reduction of the medulla volume, as reported in ant and termite species^54,55^. Furthermore, we highlight that the dimorphism of the optic lobe extends to all three neuropils and is not bound to the medulla only.

Second, female *E. dilemma* bees, from the time of emergence, possess larger mushroom bodies than males. The size increase of this neuropil was accompanied by a larger volume of Kenyon cells, the intrinsic neurons of the mushroom bodies, indicating a higher neuron number in females (Fig. 5B, D). This may be correlated with an increase in cognitive abilities of females associated with their degree of social behaviour, in which each individual gains a wider behavioural repertoire without the benefits of distributed cognition observed in advanced eusocial species^56^.

Our analysis revealed that the eyes, as well as all components of the optic lobe —the lamina, medulla, and lobula —are relatively enlarged in *E. dilemma* males (Fig. 3). This region of the brain is the primary processing centre for visual sensory input from the compound eyes^24^ (see Table 1 for further reference). Higher investment in the visual system of males has been reported in other bee species. For example, in bumblebees sexual dimorphism of the visual system is seen in species where males show perching strategies but is absent in those that patrol scent routes, as they lack a visual mate detection strategy^57^. In honeybee species, males have also been reported to have specialized visual adaptations to detect the queen bee during the mating flight and perform chasing behaviour, which involves the ability to perceive small moving objects against the sky^52,58^.

We suggest that the larger size of the optic lobe neuropils in *E. dilemma* is not associated with the need of locating a female, since in orchid bees females are the active seekers of males following perfume display^19^. Instead, we propose that this higher investment in visual areas of males is likely related to two factors: (1) males do not exhibit nest fidelity like females do, and therefore spend much of their lives moving though the landscape and experiencing novel visual landmarks. (2) Males often display at perch sites and must quickly react to the appearance of a female ready to mate or to the presence of another intruder male and engage in a stereotypical male-male completion behaviour. The optic lobe is known to facilitate attention-like processes prior to a behavioural choice^59^. Attention like processes are known to drive selective attention and in *Drosophila* it has been shown to restrict perception to prominent visual stimuli while supressing less relevant competing cues^60^. We argue that this process might be enhanced in male orchid bees to efficiently make decisions in novel landscapes and the frequent interactions with other bees (males and females) approaching display sites. Moreover, perfume collection behaviour requires the visitation of multiple and sparse environmental perfume sources^61,62^. We speculate that these sex-specific behaviours selected for enlarged optic lobes that enhance attention-like processes and/or increased resolution, however this needs explicit testing.

Although the differential expansions of brain regions can reveal sites of adaptive behaviours, not all neural adaptations show a morphological signature given the co-option of brain structures than can be adapted with minimal tuning of the underlying neural circuit^3^. Besides the differences and singular similarity in integrated regions between the sexes, most correlations within each sex were non-significant (*p* > 0.05). This suggests some developmental independence between regions and lends support to the mosaic brain hypothesis which suggests selective forces can be region-specific, having an adaptive response relatively independent of changes in other regions^63,64^.We further show that exploring the covariation of brain regions can be a useful approach to reveal sensory adaptations. For instance, we found a strong and positive morphological integration within the optic lobe, and between the optic lobe and central complex in males, with an average Pearson correlation value of 0.83 and 0.9 respectively (Fig. 6A). However, this correlation was absent in females (Fig. 6B). This male-specific trait covariation suggests a synergistic function between the primary visual processing centre and the central complex, a high-order brain region. The central complex is an ancient brain region conserved across arthropods, which evolved close to the bilaterian ancestor over 550 million years ago^65^, and is thought to be the navigation centre of the brain, coordinating complex guidance strategies, including those that rely on the sky-compass navigation^41^. Morphological integration in the brain has been shown to require the coordination of neurodevelopmental processes^66^ and is thought to evolve between regions that share a functional role^8,9^. Thus, our result of integration of visual processing centres in the male brain suggests that males not only have higher sensory demands on the visual system, but also require unique cognitive abilities related to their visually guided locomotor control.

We also found that males show an expansion in the ocellar synaptic plexi and the dorsal rim area of the compound eye (Fig. 4). These two visual regions are involved in the sensing and processing of polarized light^31,32,47^. Polarized light information is known to be important for sky-compass navigation and flight stability in bees^67,68^. In orchid bees the ocelli have been shown to facilitate integration of polarized light for navigation, with regions sensitive to both polarized and focused light^68^. This dual function, which is not the typical function of ocelli in all insects, has been proposed as adaptive for the navigation of environments with varying light conditions, as is the case of rainforests where light varies drastically with location^69^, as well as for visual discrimination. The correlated increase in the investment of neuropils involved in the primary processing of information from both the image-forming and light-capturing eyes strengthens the idea that males invest more heavily on visual processing than females.

Pronounced sexual dimorphism was also seen in the mushroom body, a well-studied high-order processing centre in the arthropod’s brain^38,70^. In *Euglossa dilemma* females, this neuropil was enlarged in both the input and output regions (Fig. 5, supplementary Table S2), with a notable enlargement of the lip, the olfactory subcompartments of the calyx^40^ (Fig. 5C). This expansion is associated with an increase in the volume of Kenyon cells (Fig. 5D), a common proxy of neuron number in comparative studies^71,72^. Although at broader evolutionary timescales, the overall expansion of mushroom bodies in Hymenoptera has been linked to the evolution of parasitoidism rather than sociality^73^, more discrete changes in their relative volume at smaller phylogenetic levels, such as sister species and closely related species, have been interpreted as sensory adaptations associated with increased cognitive demands of social organization^56,74^.

In insects, there are multiple levels of social complexity, and *E. dilemma* females have been categorized as primitively social, with small cooperative colonies commonly formed by one dominant mother and two subordinate daughters^13,75^. Under this level of social organization, the benefits of distributed cognition seen in advanced eusocial species are unlikely to emerge. As outlined in the review by Godfrey and Gronenberg (2019), the expansion of the mushroom bodies can be explained by an increasing complexity of social behaviour until advanced division of labour is achieved, since at this level individual bees tend to have narrower behavioural repertoires and reduced cognitive demands per individual. Thus, we speculate that the relative expansion of the mushroom bodies in *E. dilemma* females corresponds to this intermediate level of social organization, in which individual females exhibit a broad range of behaviours, that include starting a nest as a solitary foundress, followed by a period of low activity as a guard, followed by establishing hierarchical social organization, with dominant and subordinate roles. We speculate that the strong cognitive demands imposed by this wide range of behaviours resulted in the female-specific expansion of the mushroom bodies. Comparative studies of other bee species with simple social structure have also reported an expansion of the mushroom bodies at similar levels of sociality. For example, in closely related species of *Augochlorella* sweat bees, the loss of sociality correlates with a decreased relative volumetric investment of the mushroom body^76^. On the contrary, induced sociality in the sweat bee *Megalopta genalis* has been shown to cause an increased investment on the mushroom body, and it is suggested to be an adaptive response to the cognitive demands of social interactions^77^.

The mushroom body has been described as the prime centre for olfactory learning and memory across insects. In Hymenoptera, this region has also evolved as a multisensory region important for odour discrimination and learning^39^ as well as visual learning and memory^78^. A theoretical modelling of the mushroom body circuitry showed that an increased number of Kenyon cells increases logarithmically the capabilities of visual memories providing an ecologically relevant function^15^. This idea is further supported by the female-specific morphological integration between the mushroom body and the lobula and medulla, but not the lamina (Fig. 6B). The former two are the only primary optic neuropils with a sensory pathways conveying information to the mushroom body^79^. This would suggest that females invest more in associative learning visual landmarks needed during central place foraging and navigation. The lobula and medulla are known to provide information about colour and shape^26,27,80^ which are relevant floral cues used by bees to associate foraging patches with food (i.e. nectar and pollen) and resin for nesting material^81^.

Since females of *E. dilemma* are primitively social—meaning most transition from a solitary to a social lifestyle, except those that remain in their natal nest after emergence—these behavioral shifts are known to be accompanied by changes in brain gene expression^13^. These transitions might come with structural changes in the brain that can help locate candidate sites involved in supporting such behavioural changes. For example, the potential reduction of the optic lobes might correlate with the change from active foraging to a guarding phase, in which bees significantly reduced their time outside the nest. Comparatively studying these patterns of brain plasticity during behavioural transitions, can further elucidate on the ability of the brain to adapt to new behaviours, and potentially assess if this ability is sexually dimorphic or just context dependent. For example, if females do indeed reduce the size of their optic lobes during these transitions, would a male brain respond equally to an absence of foraging?

Given that perfume collection is directly linked to male fitness^19^, it was interesting to find a lack of a significant differences in the volumes of exclusively olfactory regions (antennal lobe and lateral horn). However, the antennal lobes did show a trend to be significantly larger in males (relative antennal lobe volume -Males: 0.031 ± 0.001; Females: 0.030 ± 0.002, *p* = 0.055). Brand et al. (2018) showed that males and females do differ in the composition of their antennal lobes. While males have a sex-specific macroglomerulus, females have more glomeruli which form a sex-specific cluster. These structural differences highlight that the although the relative size of certain brain regions can help infer behavioural adaptations, sensory specializations can be hidden in more subtle differences at the level of neural network connectivity. We propose that to further assess olfactory specialization between the sexes, a study looking at the neuroplasticity of specific glomeruli can help clarify the relationship between sex-specific behaviours and the dimorphic regions of the antennal lobe.

In this study we document dimorphic morphological integration, and the sex-specific patterns further support the volumetric morphological differences as sex-specific sensory adaptations under an ecological framework. Although the differential expansion of brain regions in insects can be used to suggest functional and evolutionary adaptations, we argue that morphological studies of insect brains can benefit from using morphological integration. The overall link found across our integration results with the reported physiological functions of the different brain regions, and the known ecology of each sex, demonstrates that trait covariation can be useful to reveal otherwise unknown relationships between brain regions and causal links between brain architecture and behaviour. Future studies may investigate the potential link between the sexual dimorphism in neuropil investment we identified and corresponding differences in sensory, cognitive and behavioural capacities of males and females, as well as an evaluation of neuroplasticity processes caused by age and experience, to fully uncover the implications of this study.

## Materials and methods

### Field collection, laboratory rearing and sample Preparation

Wild *Euglossa dilemma* nests were collected from Flamingo Gardens, Fern Forest Nature Centre, and Tree Tops Park, in Davie, Florida, USA. Nests were obtained following a published protocol^13^.A total of 25 nests were shipped overnight to UC Davis and placed in a rearing room at 25° C under a 12/12 light-dark cycle. To control for the effect of both age and experience-dependent neuroplasticity, given the high degree of post-eclosion volumetric expansion in different neuropils, all bees used in this study were less than 36 h old, monitoring the nests once a day during the morning for newly emerged bees. Each nest contained an average of 8 brood cells and to control for nest effect we aimed to collect only one individual per nest. However, due to death rates of brood cells, in two cases a male and a female were obtained from a was taken, except for one male and one female that were obtained from a nest.

Emerged bees were sexed by the morphology of their hindlegs, and immediately anesthetized by cooling to 4 ° C, followed by decapitation and subsequent fixation. Each bee was fixed in individual 1.5 ml Eppendorf vials with formaldehyde alcoholic acetic acid (also referred as FAA) for 24 h at room temperature. FAA was prepared in the lab, using the standard ratios of 10:50:5:35 of Formaldehyde, 95% ethanol, acetic acid, and distilled water respectively, and stored at room temperature. A total of 12 males and 13 females were studied.

### Sample Preparation and standardized staining

Although micro-CT scanning has been used recently for the visualization of insect brains^82–84^, the current published method which employs phosphotungstic acid as the staining agent^85^ failed to correctly stain the brains of *E. dilemma.* Here, we share a novel methodology for the visualization of soft-neural tissue in arthropods, as the method was further tested in syrphid flies and megalopas (crab larvae) to test its effectiveness (supplementary Figure S5). The method here described uses iodine, which has been a standard contrasting agent for X-ray based imaging for at least a decade, but with a new concentration, incubation times and temperature. Additionally, it involves a fixation step in formaldehyde alcoholic acetic acid prior to contrast enhancement, which proved to increases the homogenous absorption of the iodine and the signal: noise ratio during scanning. This provided a clear two- dimensional stacks along the three axes: XY (coronal plane), XZ (axial plane) and YZ (sagittal plane), (supplementary Figure S6, supplementary video 1, 2, 3). This new protocol allows for a non-destructive soft-tissue enhancement, as iodine is a smaller molecule than PTA, which diffuses through the chitin cuticle or arthropods preventing the need of an incision to the head, as needed in the protocol described by Smith et al., 2016^85^.

After the collection of samples and subsequent fixation in FAA for 24 h, the thorax width, also known as the intertegular distance, (Fig. 2A), was measured as a proxy of body size to the nearest 0.01 mm^86^. Samples were then transferred to individual containers with 80/20% ethanol/water solution until staining. For staining, we used resublimed iodine crystals (Spectrum Chemical Mfg. Corp, New Jersey) to prepare a 1% iodine solution in 100% ethanol. Staining solution was always prepared the day before samples were submerged in the solution. Each sample was individually placed in 1.5 ml glass vials and fully covered with the iodine solution for 48 h at room temperature in the dark. Sample were then washed twice for 30 s in 100% ethanol and stored in 100% ethanol to prevent iodine diffusion. To standardize image contrast, we spaced out staining, so each sample was stained the same week of their imaging. It is worth noting that staining still proved to be efficient even after one month and neural tissue showed shrinkage. This was confirmed by scanning a sample after one month of staining and registering it with the original scan using the free academic license of the Dragonfly software, Version 2022.2 for (Windows Microsoft, Redmond, Washington). Comet Technologies Canada Inc., Montreal, Canada; software available at https://www.theobjects.com/dragonfly.

### Micro-CT scanning

Bee brains were imaged at the Centre for Molecular and Genomic Imaging (CMGI), University of California, Davis, USA, using a MicroXCT-200 CT specimen scanner (Carl Zeiss X-ray Microscopy Inc., Pleasanton, USA). During scanning samples were kept in 100% ethanol. To optimize scanning times, 5 head cases were stacked in 5 mm diameter plastic straws and separated by a thin layer of cotton (supplementary Figure S7). To maximize resolution, a 4.0X objective was used and the source and detector were placed at -25 and 7 mm respectively from the sample, achieving a voxel size of 5.24 μm. Imaging was done under a beam power of 40 kV/8 W with an LE2 filter, and a bin 2 imaging with 1 s exposure was carried out to improve signal: noise ratio. A total of 3200 projections per bee were acquired, and the samples were rotated 360° during the scan, with a start angle of -180°, and an end angle of 180°. All projections were used for image reconstruction and a smoothing filter of 0.5 kernel size was applied to decrease image noise caused by drift after scanning. Reconstructions were done using *Zeiss XM Reconstructor* software, Carl Zeiss Microscopy GmbH, Oberkochen, Germany, to produce TXM files.

### Image processing and segmentation

All TXM files were exported as a 16-bit raw tiffs containing all projections and imported for data analysis to the free academic license of the Dragonfly software, Version 2022.2 for (Windows Microsoft, Redmond, Washington). Comet Technologies Canada Inc., Montreal, Canada; software available at https://www.theobjects.com/dragonfly.

To obtain individual brain volumes (µm^3^) for each sample, raw scans were first manually re-oriented in the 2D views (XY, XZ, YZ) to achieve the natural axis-orientation using six degrees of freedom with the functions Translate/Rotate. For each sex, the first bee scan of each sex was used as the fixed image to align and register all other scans (supplementary Figure S8). Samples were first manually aligned one at a time, following automatic rigid image registration with linear interpolation, and using the mutual information option for similarity metric. After all raw scans were aligned, segmentation was performed on nine main brain regions (Table 1), as well as the remaining central brain (rCB). Segmentation involved digitally isolating each neuropil as a three-dimensional region of interest that could be visualizing and analysed independently from the rest of the scan. The rCB was all the unsegmented areas of the brain after all neuropils of interest had been segmented out. In this study, we considered the central complex as a single region that includes the protocerebral bridge, fan-shaped body, ellipsoid body and noduli. For the mushroom body we further assessed the calyx as the input regions and the pedunculus and lobes as the output regions, as well as the lip and the collar, the two single-sensory subcompartments of the mushroom body calyx to evaluate specific differences in the olfactory and visual high-order processing respectively. The volume of the Kenyon Cell cluster was additionally segmented as a proxy of neuron number.

All segmentation was done using the manual circular paint brush and the semi-automatic grid tools with a 64 × 64 grid size and varying sigma values. Each neuropil was segmented as a region of interest (ROI), and a smoothing of 7 points kernel size was applied to each segmentation to standardize noise reduction prior to extracting final volumetric measurements found under the statistical properties of each ROI.

### Standardizing sex-specific brain atlases

Following image registration and segmentation of individual samples, the standardized brain atlas was achieved in Dragonfly ORS by segmenting a generated average sex-specific raw scan. To achieve this, we first standardized intensity values of all scans by normalizing histograms using the option available in the Dataset pop-up menu. After the standardization of pixel value distribution across samples, we created the average raw scan using the arithmetic function available under advance image processing. This function allows the user to set a specific function that will be applied to a series of datasets in pixel-by-pixel fashion, such a way that the output image depends entirely on the input images while conserving image spacing. For each sex, we specified an arithmetic mean function containing all samples by setting the input option to 13 for females and 12 for males and selected the “Create Dataset” option as output. This operation allowed us to create one data set per sex that was the average of all samples previously registered. The segmented brain regions were then the representation of the standardized sex-specific brain atlas, and each ROI was exported as a mesh file to help with visualization.

### Statistical analysis testing for sexual dimorphism

All statistical analyses were performed in R v.4.2.2 (R Development Core 295 Team, 2008). To assess sexual dimorphism, we tested for differences in the allometric scaling of each neuropil of interest (Table 1) by comparing them to the rCB as the allometric control, and whether the scaling deviated from isometry. The rCB was chosen as an independent measure of brain size following previous published work in other insect species^5,87^. If no significant difference was found between each pairwise comparison of the scaling coefficients, we further tested for differences in the relative investment on a given brain region by evaluating “grade-shifts” along the Y-axis in the relationship between the two variables. For such analyses we performed standardized major axis regressions (SMA) in smatr v.3.4-8^88^ using the standard allometric scaling relationship: log y = β log x + α, where β represents the allometric scaling (i.e., slope), and α represents the “relative investment” on the dependent variable (i.e., intercept), with larger α representing greater investment. All regressions were performed using the log 10-transformed absolute volumes of each ROI. Deviations from isometry were tested independently for each sex, with the function *slope.test = 1*, and evaluated with a corresponding p value and r statistic. For differences in scaling and investment, significance was evaluated with a p-value and its associated likelihood ratio and Wald statistic, respectively. We also include the sex-specific effect size for each SMA, reported as the correlation coefficient (r). The coefficient was calculated as the square root of the r^2^ value associated to each regression. The interpretation of effect sizes followed Cohen’s categorization, with *r* < 0.5 interpreted as a “large” effect, 0.3 < *r* < 0.5 as “medium” and 0.1 < *r* < 0.3 as “small”^89^. For the complete summary statistics see supplementary Table S2.

The absolute Kenyon cell volume and the absolute surface area of the mushroom body calyx was tested to assess if differences in the size of the mushroom body correlated with an increase in neuron number (under the assumption that the compression pressure of the cells, and the average size of Kenyon cell bodies is shared between sexes) and an increase in surface area of the calyx cup, in which the Kenyon cells reside. As both, the Kenyon cells volume and the calyx surface area deviated from normality (Shapiro-wilk 0.0001 and 0.01 respectively), we performed a Mann-Whitney U test to test for significance.

Additionally, we evaluated differences in the relative size of the dorsal rim area (DRA) using SMA. In orchid bees the DRA is recognizable in micro-CT scans as a protrusion with a thinner lens, so we created a width threshold on the cornea to separate the DRA from the non-DRA part of the compound eye, setting a maximum DRA cornea width corresponding to the wider part of the cornea at the start of the protrusion (supplementary Figure S4). This allowed for quantitative and repeatable approach during segmentation. Here, we use the remaining compound eye (rCE), as the allometric control. All final plots were created using the *ggplot2* R package^90^.

Finally, we evaluated the allometric scaling between the total brain size (µm^3^) to body size (µm) to test the overall investment on neural tissue. To estimate body size, our predictor variable, we used the intertegular distance known as the linear measurement between the point of attachment of the wings to the body^86^. Scaling was evaluated by fitting a sex-specific SMA to log-10 transformed data. In this case, isometric scaling was indicated by a slope of 3, to account for the different dimensionalities of the variables.

### Sex-specific patterns of morphological integration

To estimate the strength of interaction and independence between the different processing centres of the brain, we evaluated morphological integration between the different neuropils shown in Table 1. To control for the effect of size, we ran correlations on the residuals of the SMA of each neuropil. The use of residuals was implemented as our data set consisted of measured traits of continuous morphological variables, and we verified all allometric scaling across neuropils were shared between males and females, eliminating the possibility of underestimating effect sizes^91^. Correlations were then calculated using the R package *corrr*^92^. Additionally, to verify the robustness of our analysis we calculated partial correlations between neuropils using their log 10-transformed absolute volumes. For consistency we treated the rCB as our third variable to account for ‘size’ and test the analytical convergence between both approaches. This analysis was ran using the R package *pp-cor*^93^. Both methods proved to provide near identical results, yet here we use the correlation matrix from residuals for subsequent analysis, as partial correlations are more sensitive to produce typo II errors with small sample sizes^94^. All correlation matrices were then plotted with *ggcorrplot*^95^. Partial correlation matrices can be found in supplementary Figure S9.

## Electronic supplementary material

Below is the link to the electronic supplementary material.

Supplementary Material 1

Supplementary Material 2

Supplementary Material 3

Supplementary Material 4

Supplementary Material 5

## Data Availability

The findings of this study are supported by data provided in the supplementary information files, and files available in the Figshare repository under the following link: https://figshare.com/s/98d44565e196f20956ea.
