# Supplementary material for: Sexual dimorphism and morphological integration in the orchid bee brain
Source: Sci Rep. 2025 Mar 14;15:8915. doi: 10.1038/s41598-025-92712-3 (PMC11909157; doi:10.1038/s41598-025-92712-3)
Supplement: Supplementary file 4 — Supplementary Material 4 [file 41598_2025_92712_MOESM4_ESM.docx]

**Supplemental video legends for: Sexual dimorphism and morphological integration in the orchid bee brain**

Supplementary Video 1: Time-lapse of micro-CT scan slices on the coronal (XY) plane.

Supplementary Video 2: Time-lapse of micro-CT scan slices on the axial (XZ) plane.

Supplementary Video 3: Time-lapse of micro-CT scan slices on the sagittal (YZ) plane.
