# Supplementary material for: Sexual dimorphism and morphological integration in the orchid bee brain
Source: Sci Rep. 2025 Mar 14;15:8915. doi: 10.1038/s41598-025-92712-3 (PMC11909157; doi:10.1038/s41598-025-92712-3)
Supplement: Supplementary file 5 — Supplementary Material 5 [file 41598_2025_92712_MOESM5_ESM.docx]

**Supplemental material for:** **Sexual dimorphism and morphological integration in the orchid bee brain**

**
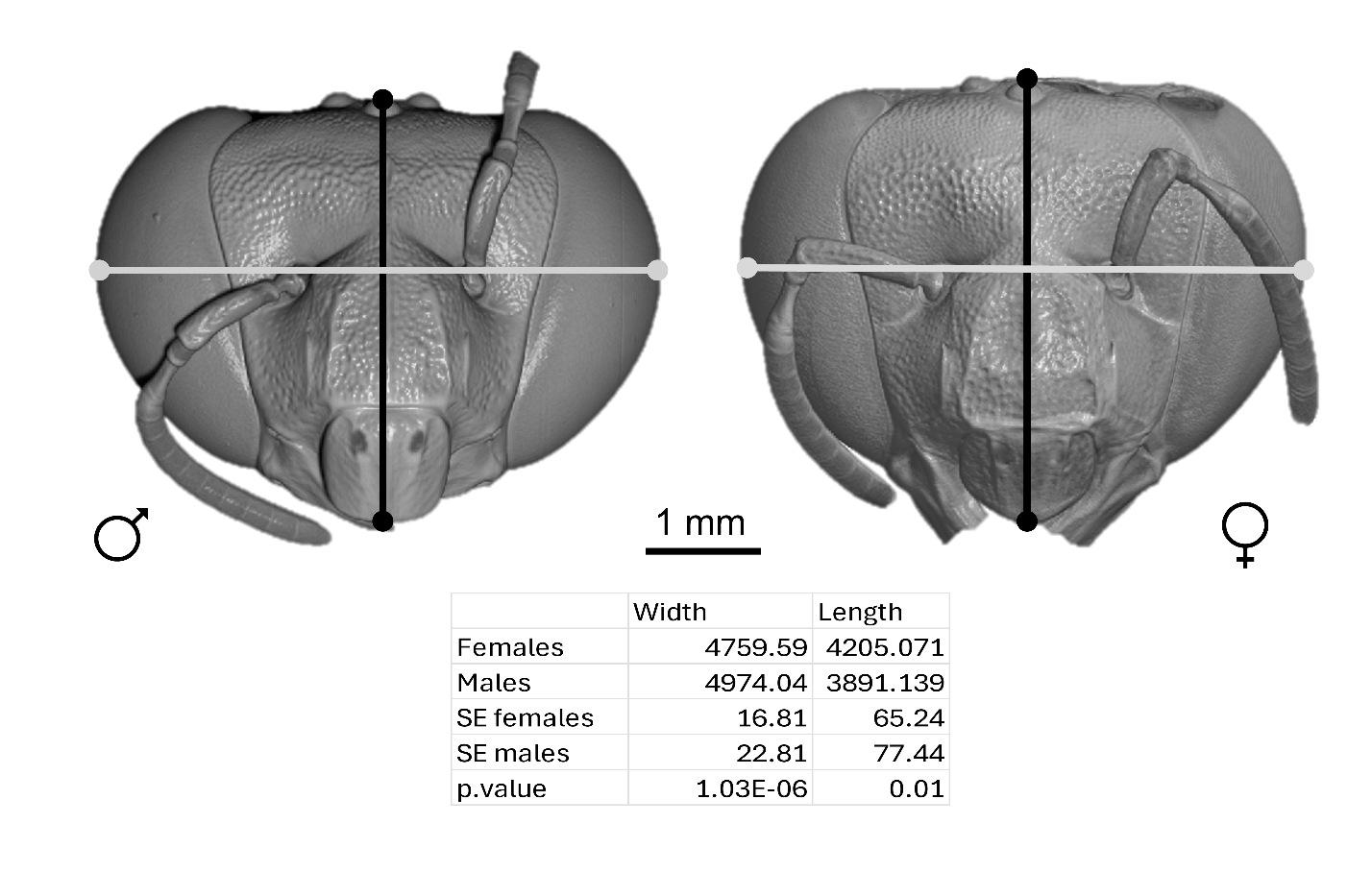
**

**Supplemental Figure S1** Summary of the length (black line) and width (grey line) of the head capsule measured for *Euglossa dilemma* males and females

**Supplemental Table S2**- Summary statistics for each standardized major axis regression

**Supplemental Table S3**-Relative increased investment of sexually dimorphic regions with fitted shared slope

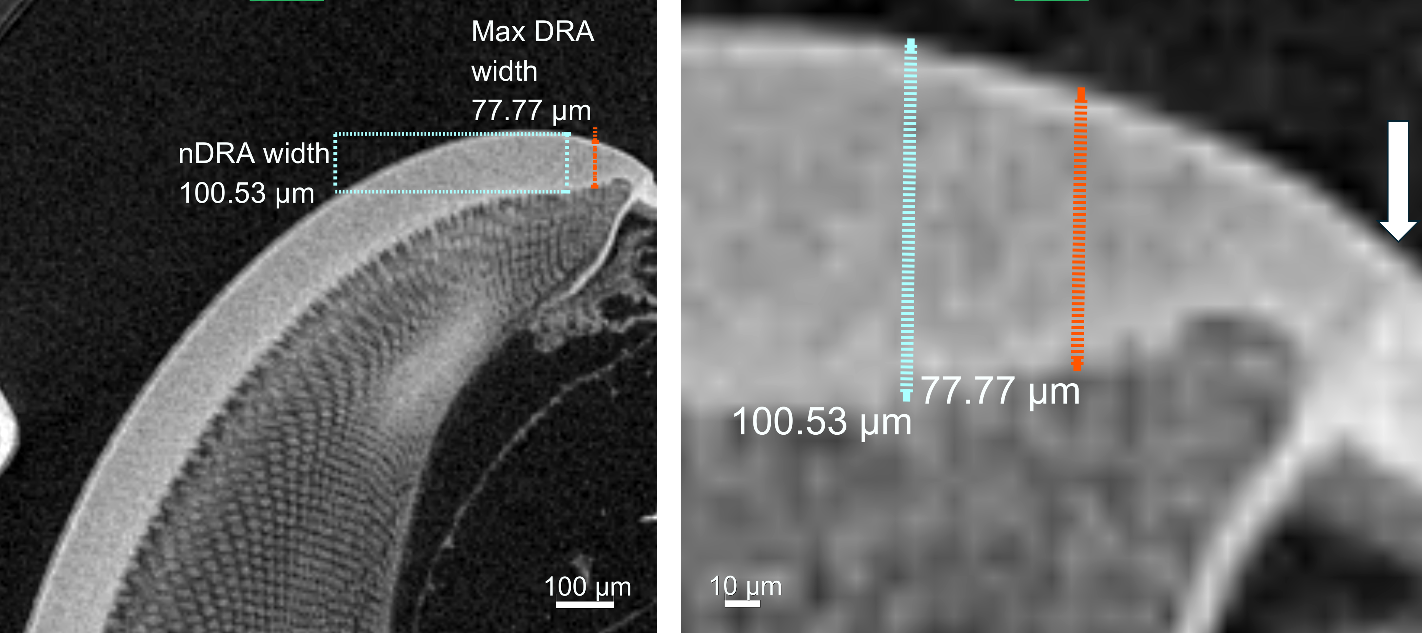


**Supplemental Figure S4** A single micro-CT slice showing the cornea thickness of the compound eye dorsal most area (aquamarine) and its thinning threshold at the dorsal most margin with a subsequent protrusion corresponding to the start of dorsal rim area, which expands from the orange line onwards to the end of the eye (white arrow)


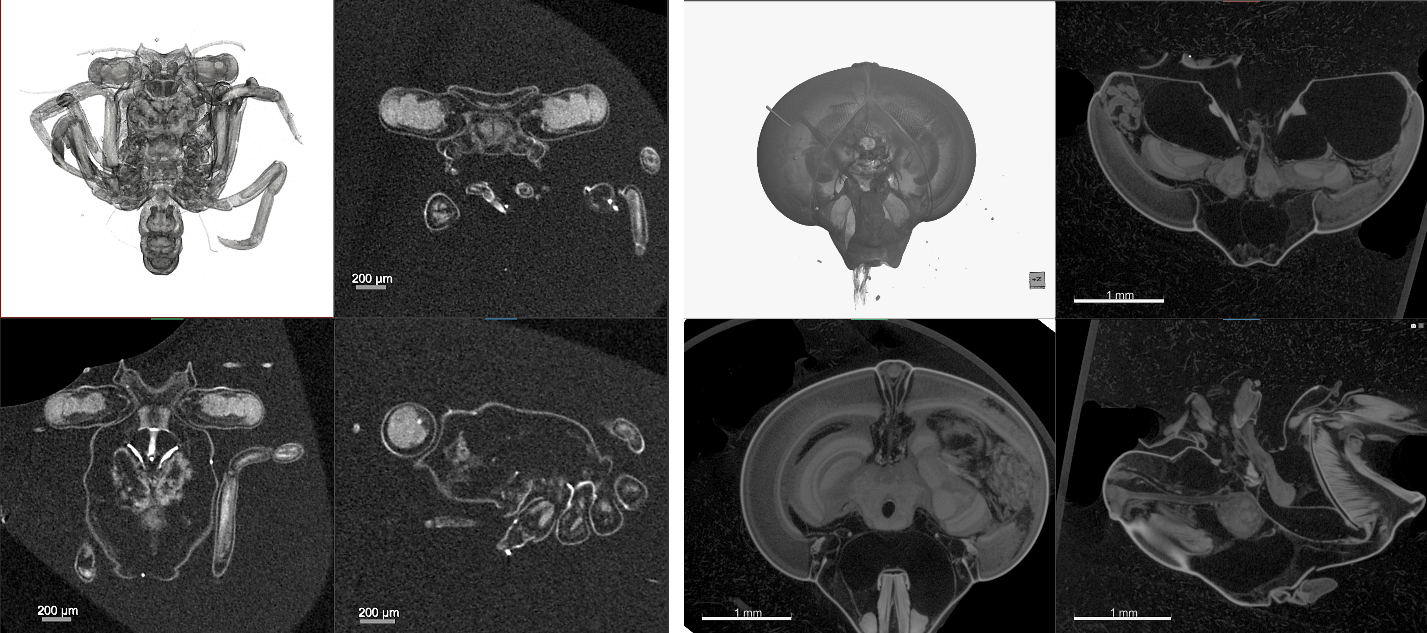


**Supplemental Figure S5.** Method testing on: Left panel: Megalopa sp .Right panel: Syrphid fly *Eristalis arbostorum* , commonly known as the European drone. Material was kindly donated by Javier Luque and Peter Coggan, respectively.

**
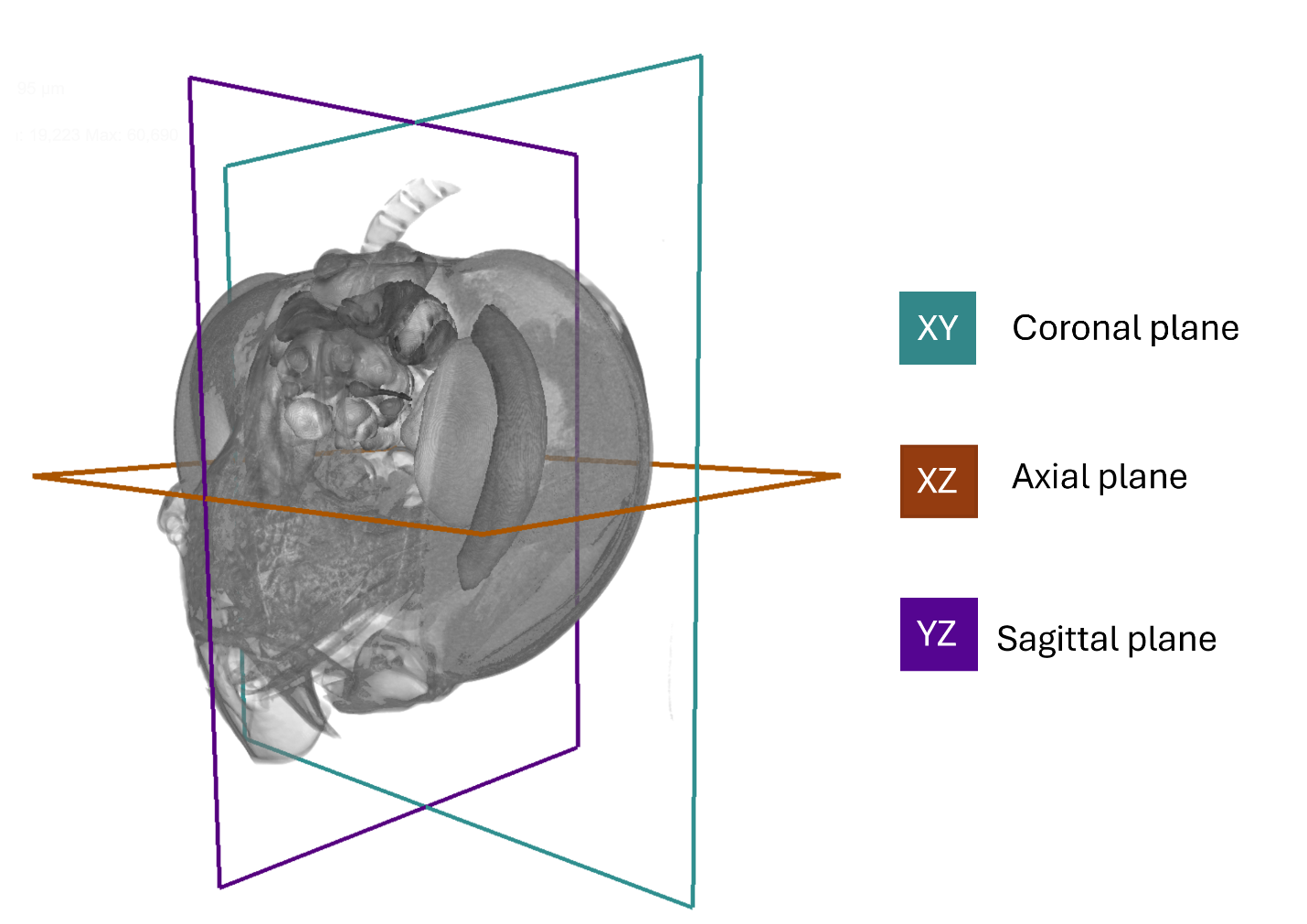
Supplemental Figure S6-** Visible planes on each micro-CT scans volume acquisition


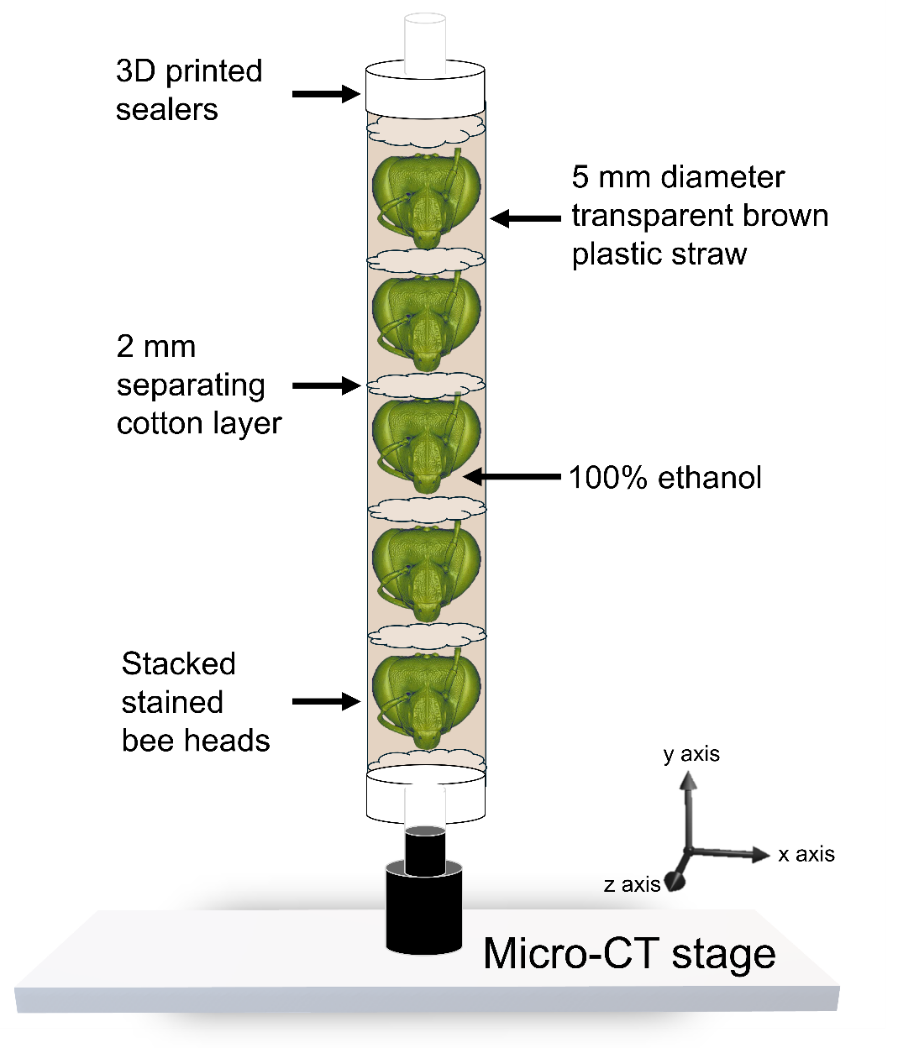


**Supplemental Figure S7**- Specimen mounting for optimized scanning times with set coordinates and automatic stage movement along the y axis


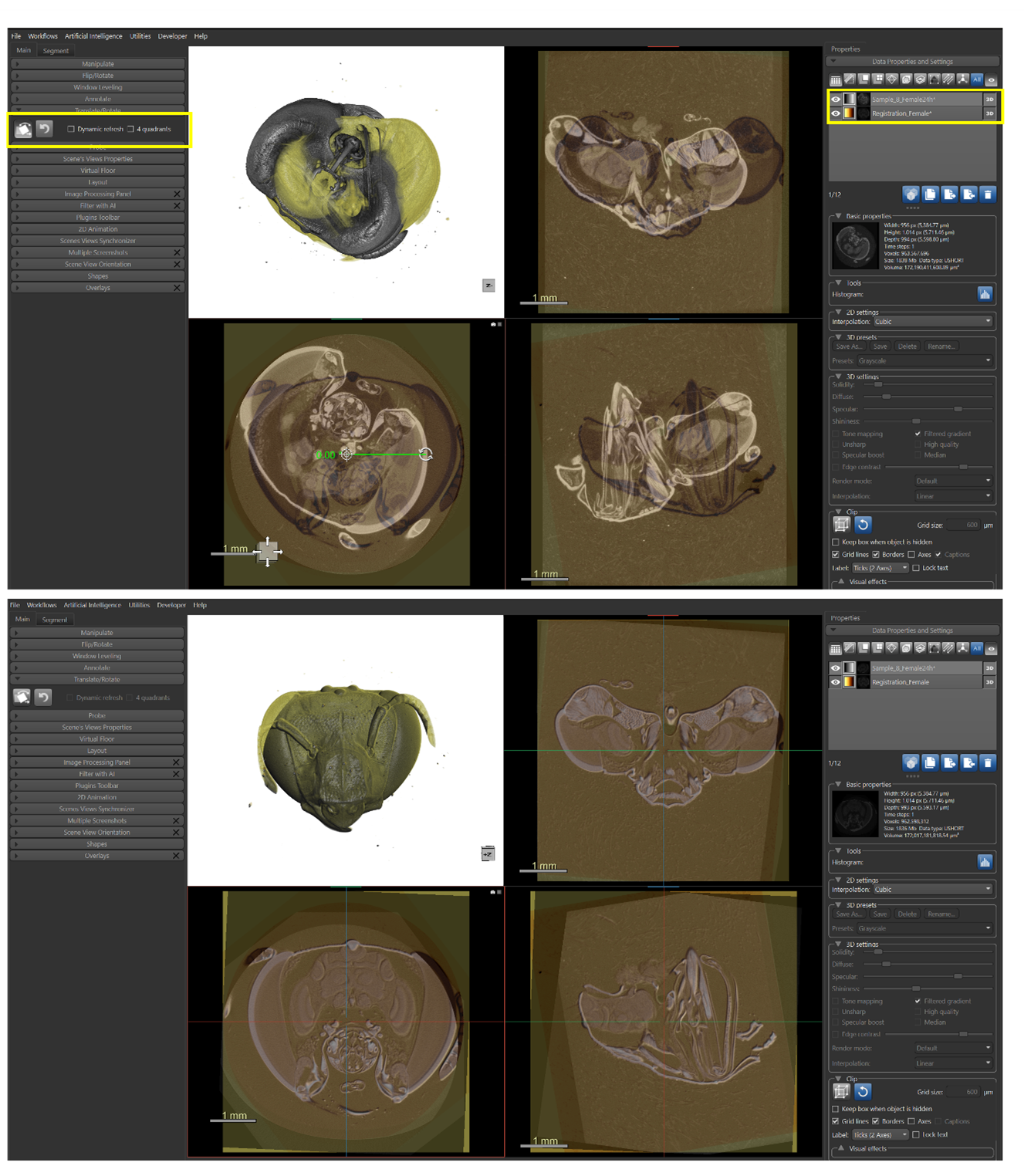
**Supplemental Figure S8**- Image alignment and registration prior to segmentation. Upper panel shows the fixed female scan template (amber color pallet), with a raw scan not yet aligned (grey color pallet). Lower panel shows the result after image alignment and rigid registration.


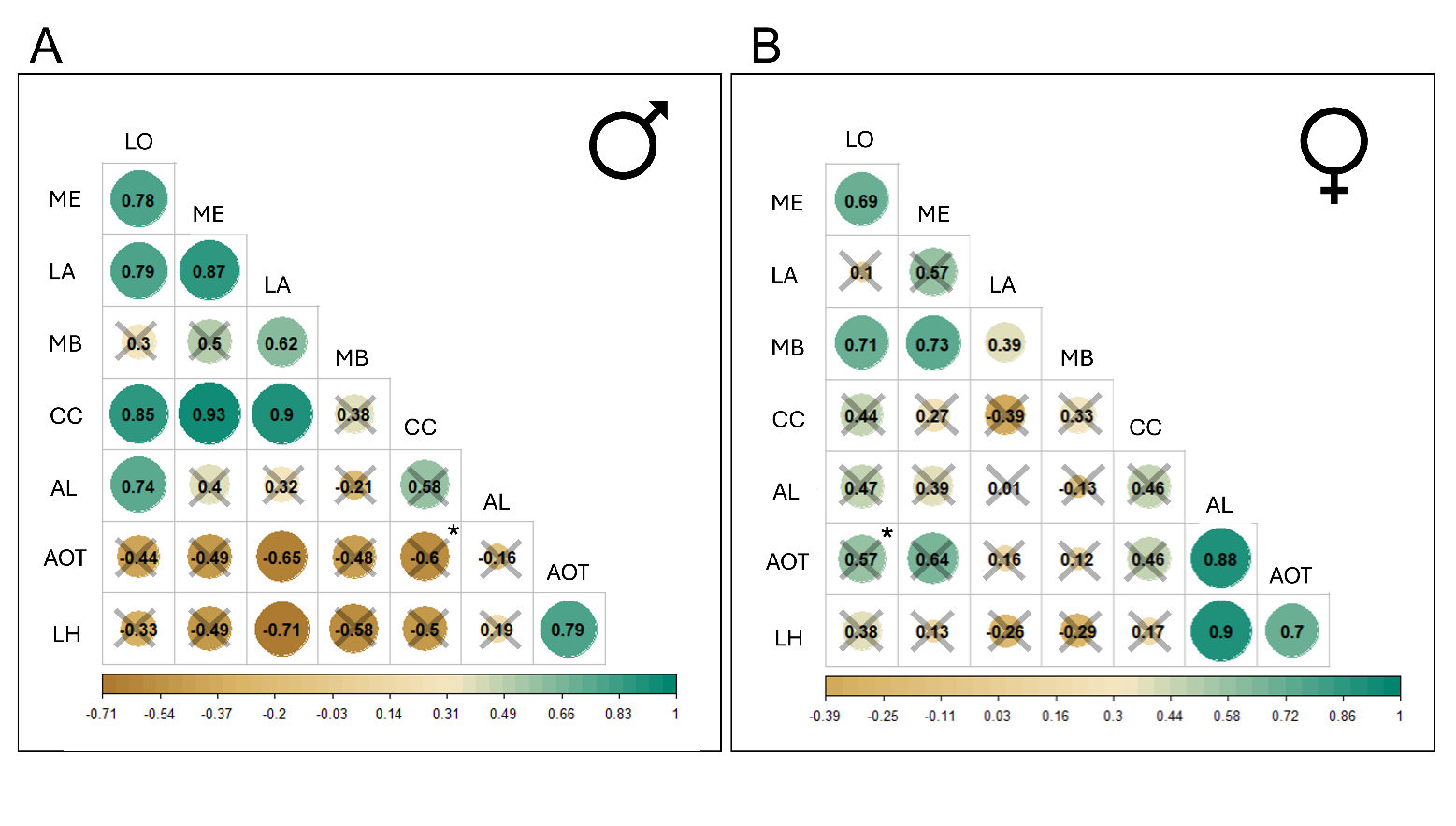
**Supplemental Figure S9**- Partial correlation matrices for *Euglossa dilemma* A) males and B) females. Non-significant correlations are crossed-out and asterisks indicate p.values close to 0.05, showing a trend towards significance
